# Preclinical Study of ^68^Ga-DOTATOC: Biodistribution Assessment in Syrian Rats and Evaluation of Absorbed Dose in Human Organs

**DOI:** 10.7508/aojnmb.2016.04.004

**Published:** 2016

**Authors:** Mojdeh Naderi, Samaneh Zolghadri, Hassan Yousefnia, Ali Ramazani, Amir Reza Jalilian

**Affiliations:** 1 Department of Chemistry, University of Zanjan, Zanjan, Iran; 2 Nuclear Science and Technology Research Institute (NSTRI), Tehran, Iran

**Keywords:** Ga-68, Octreotide, Internal Dosimetry, Somatostatin

## Abstract

**Objective(s):**

Gallium-68 DOTA-DPhe^1^-Tyr^3^-Octreotide (^68^Ga-DOTATOC) has been applied by several European centers for the treatment of a variety of human malignancies. Nevertheless, definitive dosimetric data are yet unavailable. According to the Society of Nuclear Medicine and Molecular Imaging, researchers are investigating the safety and efficacy of this radiotracer to meet Food and Drug Administration requirements. The aim of this study was to introduce the optimized procedure for ^68^Ga-DOTATOC preparation, using a novel germanium-68 (^68^Ge)/^68^Ga generator in Iran and evaluate the absorbed doses in numerous organs with high accuracy.

**Methods:**

The optimized conditions for preparing the radiolabeled complex were determined via several experiments by changing the ligand concentration, pH, temperature and incubation time. Radiochemical purity of the complex was assessed, using high-performance liquid chromatography and instant thin-layer chromatography. The absorbed dose of human organs was evaluated, based on biodistribution studies on Syrian rats via Radiation Absorbed Dose Assessment Resource Method.

**Results:**

^68^Ga-DOTATOC was prepared with radiochemical purity of >98% and specific activity of 39.6 MBq/nmol. The complex demonstrated great stability at room temperature and in human serum at 37°C at least two hours after preparation. Significant uptake was observed in somatostatin receptor-positive tissues such as pancreatic and adrenal tissues (12.83 %ID/g and 0.91 %ID/g, respectively). Dose estimations in human organs showed that the pancreas, kidneys and adrenal glands received the maximum absorbed doses (0.105, 0.074 and 0.010 mGy/MBq, respectively). Also, the effective absorbed dose was estimated at 0.026 mSv/MBq for ^68^Ga-DOTATOC.

**Conclusion:**

The obtained results showed that ^68^Ga-DOTATOC can be considered as an effective agent for clinical PET imaging in Iran.

## Introduction

Today, radiolabeled ligands play a significant role in the diagnosis and treatment of malignancies. Among carriers used for the preparation of these compounds, peptides are of particular importance due to their easy synthesis, high expression of peptide receptors in numerous cancers (compared to their relatively low density in physiological organs), high receptor binding affinity and favorable pharmacokinetic properties (1-3).

Considering the high concentration of somatostatin receptors in a wide range of human tumors, e.g., neuroendocrine, breast, lung, lymphatic and nervous system malignancies, these receptors are regarded as potential targets for peptide receptor-targeted scintigraphy (4,5). Therefore, application of DOTA-conjugated somatostatin analogues, along with suitable therapeutic and diagnostic radionuclides, is increasing in human studies (6-8).

Octreotide is a synthetic peptide analogue with a cyclic 8-amino-acid structure, developed to overcome the limitations of native somatostatin such as very low elimination half-time (9). Use of this agent has been approved in many countries for the management of hormonal symptoms in patients with gastrointestinal tumors, pancreatic neuroendocrine tumors and acromegaly (10).

Among a series of radiolabeled octreotide analogues, introduced for targeting somatostatin receptor-expressing tumors, DOTA-DPhe^1^-Tyr^3^-Octreotide (DOTATOC) indicated advantageous properties in tumor models (4) and could be labeled with a variety of radiometals (11).

Various radiolabeled DOTATOC complexes have been developed, showing high stability in human serum and significant affinity to somatostatin receptors (12). Comparative study between gallium-67 (^67^Ga)-DOTATOC and indium-111 (^111^In)-DOTATOC has indicated higher tumor and lower kidney uptakes in a somatostatin receptor-positive AR42J tumor-bearing rat model in ^67^Ga-DOTATOC (13).

The difference in the biodistribution of these complexes can be attributed to the discrepancies in the coordination chemistry of metals after radiolabeling. Previous research has shown that gallium-labeled DOTATOC is one of the most suitable somatostatin analogues developed so far (13).

Over the past decade, with the development and extension of positron emission tomography (PET) and introduction of germanium-68/gallium-68 (^68^Ge/^68^Ga) generators with suitable eluates for labeling, interest in the use of ^68^Ga has increased (14). ^68^Ga undergoes disintegration via β^+^ decay (maximum energy of 1.92 MeV) and electron capture with a short half-life of 67.8 min. Short half-life of this element makes it an appropriate candidate for nuclear medicine imaging (15).

^68^Ga-DOTATOC is the most widely applied ^68^Ga-based radiopharmaceutical (16). This new imaging agent has demonstrated high sensitivity for the detection of neuroendocrine tumors, compared to ^111^In-octeroetide as the only radiopharmaceutical approved by the Food and Drug Administration (FDA) for somatostatin-receptor scintigraphy (17, 18). In addition, the short time interval between injection and scan can be considered as another advantage of this agent.

Considering the favorable characteristics of ^68^Ga-DOTATOC in somatostatin-receptor scintigraphy, we aimed to develop an optimized method for the preparation of this novel agent, using ^68^Ge/^68^Ga generator in Iran. Besides, biodistribution of this complex in male Syrain rats was studied. Subsequently, the absorbed dose of this agent in human organs was investigated, based on the biodistribution data in rats.

## Methods

The ^68^Ge/^68^Ga generator (50 mCi/day activity) was obtained from Pars Isotope (Karaj, Iran). DOTATOC was purchased from ABX (Radeberg, Germany), and the rest of chemical reagents were obtained from Sigma-Aldrich (Heidelberg, Germany). Moreover, Whatman No. 2 paper was purchased from Whatman Company (Buckinghamshire, U.K.).

Radio-chromatography was performed, using a thin-layer chromatography scanner (Bioscan AR2000, Paris, France), and imaging studies were conducted, using a dual-head SPECT system (DST-XL, Sopha Medical Vision, Buc, France). Activity of the samples was measured by a p-type coaxial high-purity germanium (HPGe) detector (EGPC 80-200R), coupled with a multi-channel analyzer card system (GC1020-7500SL, Canberra, USA).

Calculations were based on the 511 keV peak. All values were expressed as mean ± standard deviation (mean ± SD). The obtained data were compared, using student’s t-test. P-value less than 0.05 was considered statistically significant. Animal studies were performed in accordance with the guidelines by the United Kingdom Biological Council on the use of living animals in scientific investigations (second edition).

### Elution of ^68^Ge/^68^Ga generator

For selecting a suitable eluent, the generator was eluted by 5 mL HCl, with different concentrations ranging from 0.1 to 1.0 M. Each time, the activity of the eluted ^68^Ga was measured, using the HPGe detector. Also, in order to optimize the minimum required volume for ^68^Ga elution with maximum yield and radioactive concentration, the generator was eluted with an equal volume of HCl. Afterwards, activity of each fraction, containing 0.5 mL of the eluate, was evaluated.

### Quality control of the eluted ^68^Ga

The radionuclidic purity of the eluted ^68^Ga from the generator was investigated via gamma spectrometry of decayed ^68^Ga samples. The content of chemical impurities was determined by Inductively Coupled Plasma-Optical Emission Spectroscopy (ICP-OES) method.

Radiochemical purity of the eluted ^68^Ga was studied, using instant thin-layer chromatography (ITLC) method in accordance with the reported procedure (19). For this purpose, ITLC chromatograms of ^68^GaCl_3_ solution were obtained in 10% ammonium acetate: methanol on silica gel sheets and 10 mM diethylene triamine pentaacetic acid (DTPA) solution (pH=4) on Whatman No. 2 paper.

### Radiolabeling of DOTATOC with ^68^GaCl_3_

A stock solution of DOTATOC with the concentration of 1 µg/µL was prepared in distilled water. The first fraction of the eluted ^68^Ga was discarded and the next three fractions, including 1.5 mL of ^68^GaCl_3_, were used for radiolabeling. A certain amount of DOTATOC was added to the vial containing ^68^GaCl_3_, and pH of the reaction mixture was adjusted, using HEPES.

In order to obtain the optimized conditions, several experiments were performed by changing the ligand concentration, pH, temperature and incubation time. Afterwards, 8 mL of water was added to the final solution, and the mixture was passed through a Sep-Pak C_18_ column, preconditioned with 5 mL ethanol, 10 mL water and 10 mL air, respectively. The column was then washed with 0.5 mL ethanol and 1 mL of 0.9% NaCl.

### Quality control of the radiolabeled complex

Radiochemical purity of the radiolabeled complex was assessed, using both high-performance liquid chromatography (HPLC) and ITLC methods. Paper chromatography was carried out, using Whatman No. 2 paper and various mobile phase mixtures [acetonitrile:water (1:1), 0.9% NaCl and 0.1 M sodium citrate].

HPLC was performed on the final preparation, using a C_18_ODS column with a dimension of 100×4.6 m^2^ and 5 µm particle size. Gradient elution was applied with the following parameters: water (A) + 1% trifluoroacetic acid, acetonitrile (B), flow rate of 2.6 mL/min, 100% A:0% B for 3 min, 50% A:50% B for 7 min and 0% A:100% B for 5 min.

### Stability studies

The stability of the complex at room temperature and in human serum was studied according to the conventional ITLC method. The radiolabeled complex was kept at room temperature for 120 min, while being assessed by ITLC at the specified time intervals (10, 20, 30, 45 and 120 min). For the evaluation of serum stability, 37 MBq of ^68^Ga-DOTATOC was added to 500 µL of freshly prepared human serum, and the mixture was incubated at 37 °C for 2 h; the aliquots were analyzed by ITLC method.

### Biodistribution of ^68^GaCl_3_ and the radiolabeled complex in Syrian rats

Intravenously, 100 μL of the final ^68^Ga-DOTATOC solution with approximately 5.55 MBq radioactivity was injected into male Syrian rats through the tail vein. Also, for better comparisons, biodistribution of ^68^GaCl_3_ in 0.9% normal saline (pH=7) was investigated, followed by intravenous administration of 100 μL of the solution (5.55 MBq).

The total amount of radioactivity injected in each animal was measured by counting the 1 mL syringe before and after the injection in a dose calibrator with fixed geometry. The biodistribution of the solution among tissues was determined by sacrificing four rats at each selected time interval (15, 30, 60 and 120 min) after injection, based on animal care protocols.

Blood samples were rapidly taken after animal sacrifice. The tissues (heart, kidneys, spleen, stomach, intestine, lung, liver, skin, bone, muscle, thyroid, adrenal glands, salivary gland and pancreas) were weighed and rinsed with normal saline and their activities were determined, using a p-type coaxial HPGe detector, coupled with a multi-channel analyzer, based on Equation 1 (20):


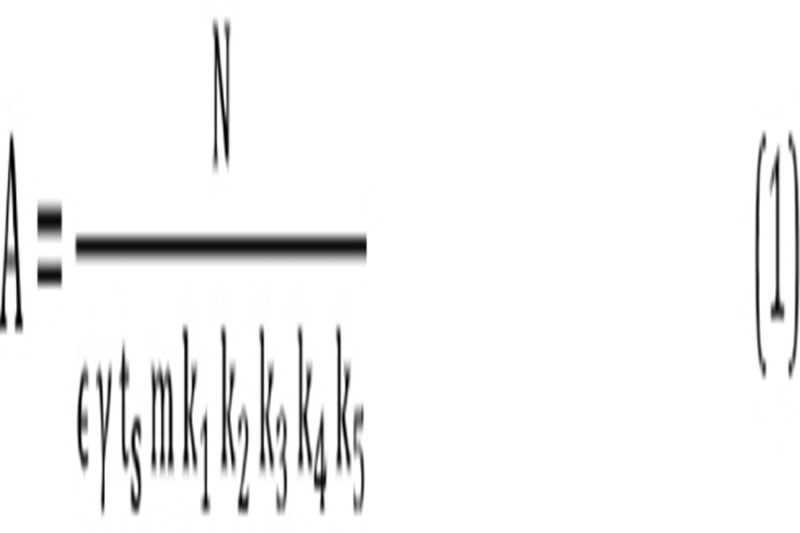


where ε is the efficiency at photopeak energy, γ is the emission probability of the gamma line corresponding to the peak energy, is the live time of the sample spectrum collection in seconds, m is the mass (kg) of the measured sample and k1, k2, k3, k4, k5 are the correction factors for the nuclide decay since the time of sample collection for starting the measurements, the nuclide decay during the counting period, self-attenuation in the measured sample, pulse loss due to random summing and pulse loss by coincidence, respectively. N is the corrected net peak area of the corresponding photopeak, calculated as:


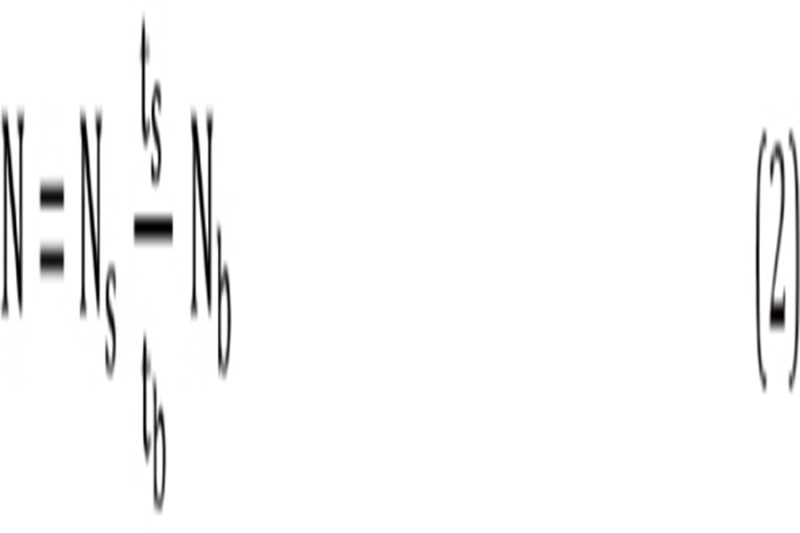


where Ns is the net peak area in the sample spectrum, N_b_ is the corresponding net peak area in the background spectrum and t_b_ is the live time of the background spectrum collection in seconds.

The uncertainty in activity measurement was calculated on the basis of the formula for error propagation, as given below:


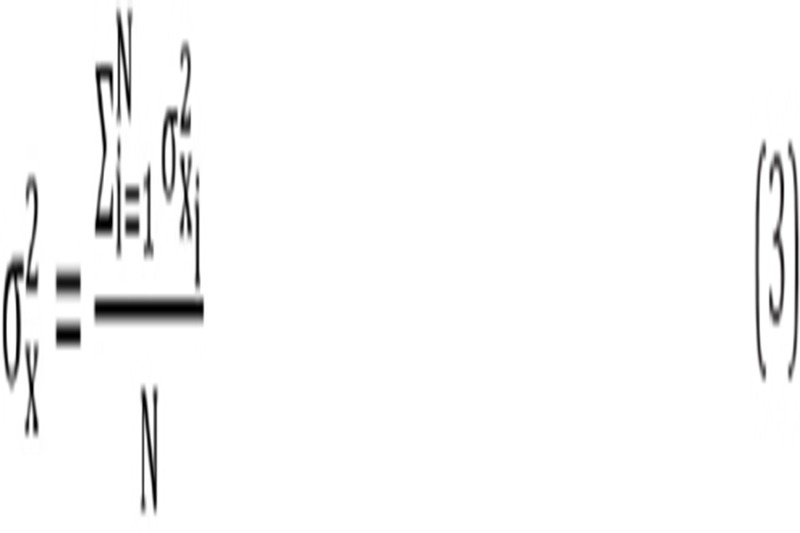


where σ_xi_ is the uncertainty in each experiment and N is the number of experiments.

The percentage of the injected dose per gram (%ID/g) for different organs was calculated by dividing the amount of activity in each tissue (A) to the decay-corrected injected activity and the mass of each organ. Four rats were sacrificed at each time interval. All values were expressed as mean ± standard deviation and the data were compared, using student’s t-test.

### Imaging studies

The Syrian rats were intravenously injected 5.55 MBq of the final ^68^Ga-DOTATOC solution through their tail vein. Planar images were obtained at the specified time intervals (15, 30, 60 and 120 min) after the administration of the radiolabeled complex by a dual-head SPECT system. The mouse-to-high energy septa distance was 12 cm. Also, the useful field of view was 540 mm×400 mm.

### Calculation of the accumulated activity in human organs

The accumulated source activity for each animal organ was calculated according to Equation 4, where A (t) is the activity of each organ at time t:


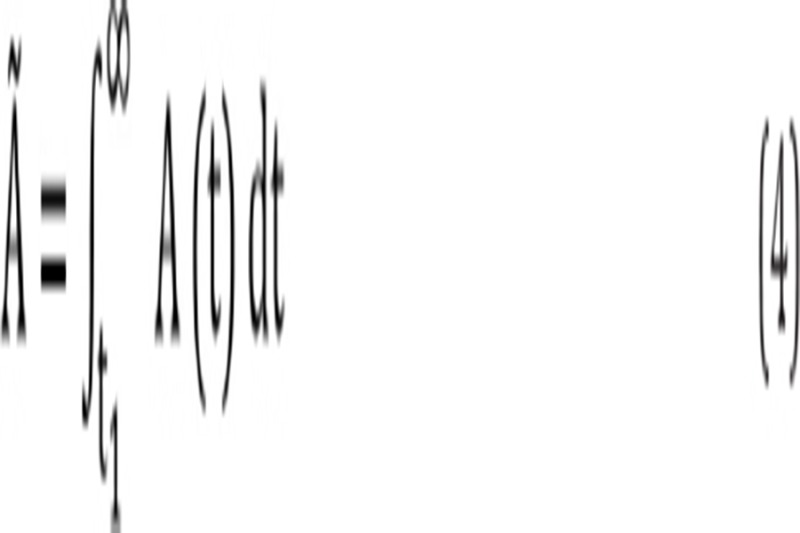


For this purpose, data points representing the non-decay corrected percentage of the injected dose were created. A linear approximation was applied between the two experimental points of time. The curves were extrapolated to infinity by fitting the tail of each curve to a mono-exponential curve with an exponential coefficient equal to the physical decay constant of ^68^Ga.

The accumulated activity in animals was calculated by computing the area under the curves, extrapolated to the accumulated activity in human organs, using a method proposed by Sparks et al. (Equation 5) and the standard mean weight of each human organ (21, 22, 23):


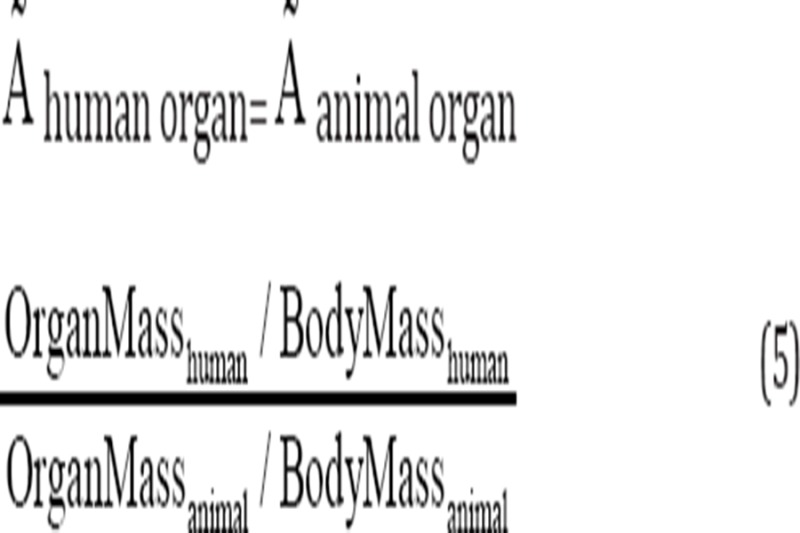


### Calculation of the equivalent absorbed dose

The absorbed dose in human organs was calculated by the Radiation Absorbed Dose Assessment Resource (RADAR) formalism, based on the biodistribution data in rats (22):


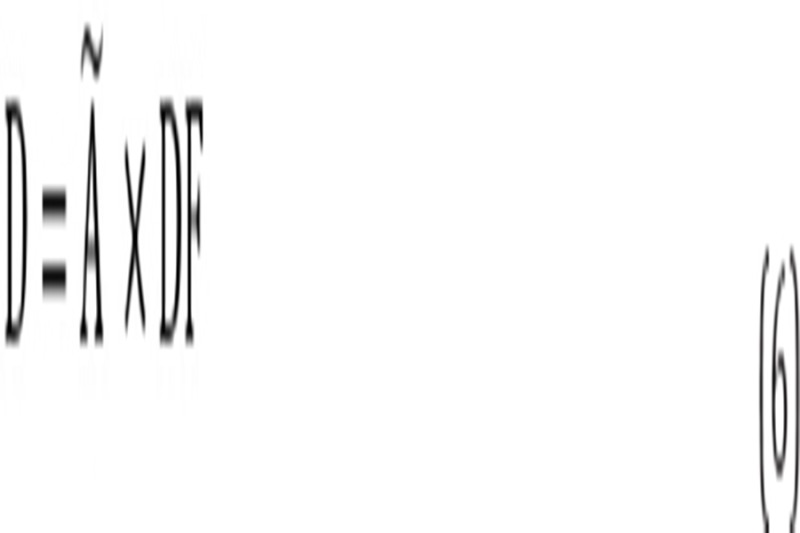


where is the accumulated activity in each human organ and “Dose Factor” is:


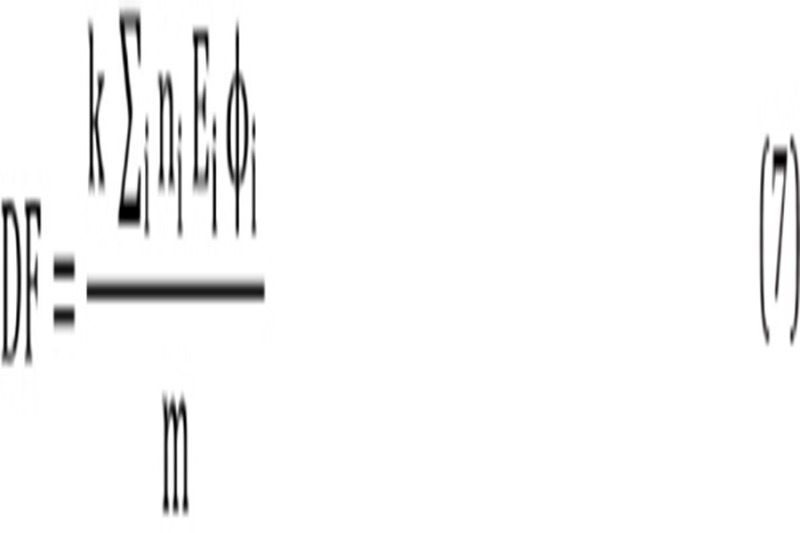


In the equation above, n_i_ is the number of radiations with energy E emitted per nuclear transition, E_i_ is the energy per radiation (MeV), Q_i_ is the fraction of emitted energy absorbed by the target, m is the mass of the target region (kg) and k is the proportionality constant 
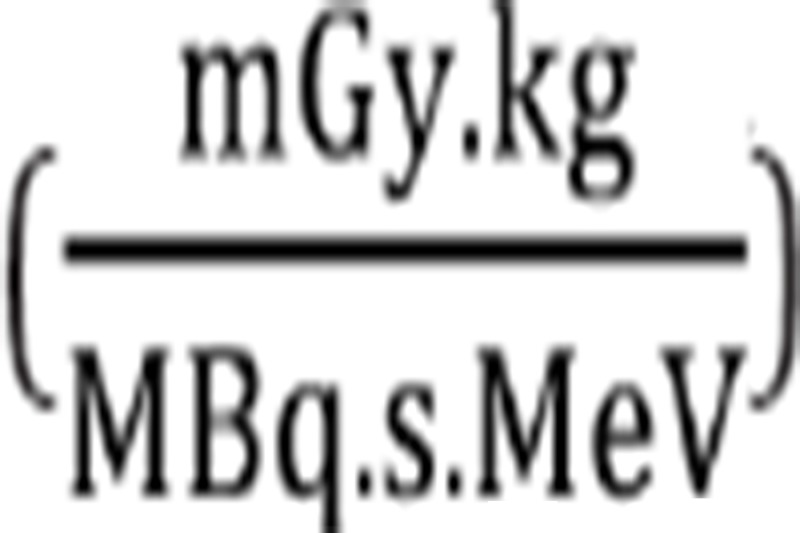
 DF represents the physical decay characteristics of the radionuclide, the range of emitted radiations and the organ size and configuration, expressed in mGy/MBq.s (24). In this study, DF values were obtained from OLINDA/EXM software (22).

### Calculation of the effective absorbed dose

The effective absorbed dose in each organ was calculated, using the following formula:


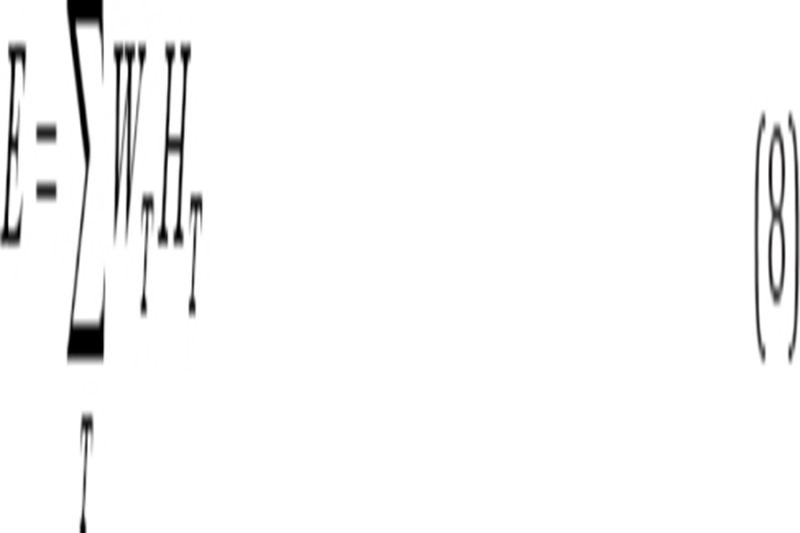


where H_T_ is the equivalent absorbed dose in each organ and W_T_ is the tissue-weighing factor, which represents a subjective balance between different stochastic health risks (25). W_T_ was obtained from the International Commission on Radiological Protection (ICRP) publication 103 (26).

## Results

### Elution of ^68^Ge/^68^Ga generator

While the generator was eluted by 5 mL HCl with different concentrations ranging between 0.1 and 1.0 M, the eluted ^68^Ga activity increased by the increment in HCl concentration. This finding indicated a higher elution yield for 1.0 M HCl. However, for radiolabeling, 0.6 M HCl was determined as the suitable solvent for the generator elution.

**Figure 1:**
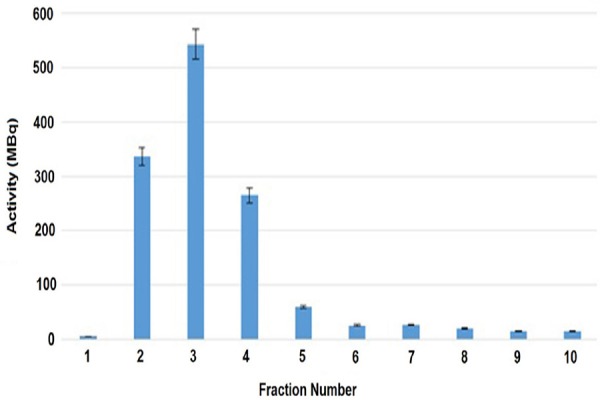
Elution diagram of the ^68^Ge/^68^Ga generator used in this study.

The activity of 0.5 mL fractions of ^68^Ga eluted with 0.6 M HCl was measured, using the HPGe detector. As indicated in Figure 1, maximum activities were obtained in the second, third and fourth fractions. Therefore, it is recommended to elute the generator with 0.5 mL HCl and dispose of the eluate; elution should be performed again with 1.5 mL HCl for further use in radiolabeling.

### Quality control of the eluted ^68^Ga

**Figure 2:**
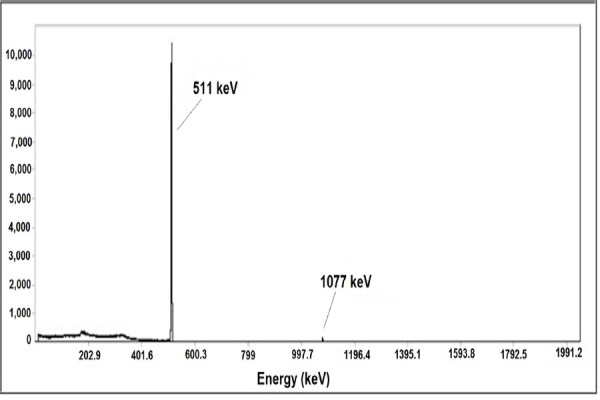
Gamma Spectrum of the eluted ^68^Ge after 48 h of the generator elution.

Radionuclidic purity was investigated by measuring the eluate in the HPGe detector for 1000 s after 48 h of generator elution. The HPGe spectrum showed the presence of 511 and 1077 keV energies, originating from ^68^Ga (Figure 2). The concentrations of tin, zinc and copper in the eluted ^68^Ga were calculated to be <0.1, 0.23 and 0.38, respectively.

**Figure 3:**
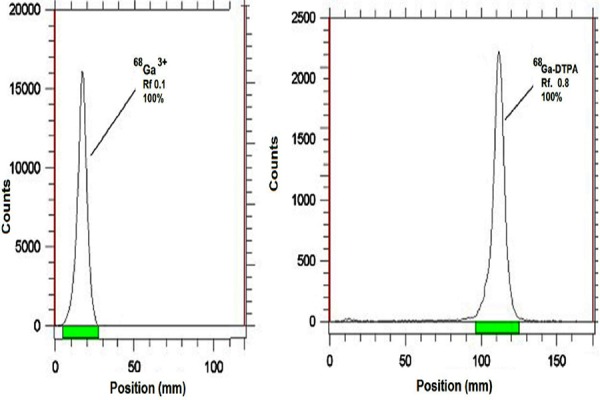
ITLC chromatogram of ^68^GaCl3 solution in DTPA (pH=5) (right) and 10% ammonium acetate: methanol (1:1) (left) using Whatman No. 2.

Radiochemical purity of ^68^GaCl_3_ solution was assessed in two solvents. Generally, in 10 mM DTPA solution, free ^68^Ga^3+^ is coordinated to a more lipophilic moiety as ^68^Ga(DTPA)^2-^ and migrates to a higher R_f_. On the other hand, in the 10 % ammonium acetate:methanol mixture (1:1), ^68^Ga^3+^ remains at the origin, while other ionic cations of ^68^Ga^3+^ migrate to a higher R_f_; however, such finding was not reported here (Figure 3).

### Radiolabeling of DOTATOC with ^68^GaCl_3_

In order to obtain the maximum radiochemical purity, several experiments were carried out by varying the reaction parameters such as ligand concentration, pH, temperature and reaction time. The effect of pH on radiochemical purity was studied by varying the pH of the reaction mixture, using HEPES. The results indicated that the optimal pH for radiolabeling was within a range of 3.5-4.

The results also indicated that even at very low concentrations of the ligand (16 µg), ^68^Ga-DOTATOC can be prepared with the radiochemical purity of more than 98%. The experiments showed that seven minutes can be sufficient for radiolabeling at a temperature of 90-95 °C.

### Quality control of the radiolabeled complex

**Figure 4:**
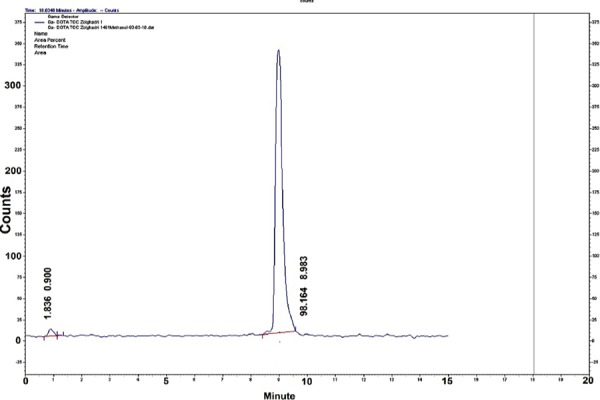
HPLC chromatogram of ^68^Ga-DOTATOC.

Radiochemical purity of the radiolabeled complex was assessed, using both HPLC and ITLC methods. HPLC analysis showed that the fast eluting compound was a hydrophilic ^68^GaCl_3_ cation (0.9 min), while ^68^Ga-DOTATOC with a high molecular weight was eluted after 8.98 min (Figure 4). The chromatogram of the final product indicated a radiochemical purity of more than 98%.

In ITLC, different chromatographic systems were applied for the detection of the radiolabeled compound from the free gallium cation (Table 1). By using Whatman No. 2 paper, along with 0.1 M sodium citrate and 0.9% NaCl as the mobile phases, the radiolabeled compound remained at the origin, while free gallium cation migrated to a higher R_f_.

**Table 1:** R_f_ values of ^68^GaCl_3_ and ^68^Ga-DOTATOC in different chromatographic systems

Chemical species	Mobile phase	Stationary phase	R_f_
^68^GaCl_3_	0.1 M sodium citrate (pH 4)	Whatman No.2	0.7
	
^68^Ga-DOTATOC			0.1

^68^GaCl_3_	0.9% NaCl	Whatman No.2	0.9
	
^68^Ga-DOTATOC			0.1

^68^GaCl_3_	Acetonitrile :Water (1:1)	Whatman No.2	0.1
	
^68^Ga-DOTATOC			0.6

On the other hand, ^68^Ga-DOTATOC eluted faster in acetonitrile:water (1:1) mixture and was detected at an R_f_ of 0.6. ITLC studies approved the production of the radiolabeled complex with more than 99% radiochemical purity. Also, the results confirmed the application of each three solvents in the ITLC analysis.

### Stability studies

The stability of ^68^Ga-DOTATOC was investigated at room temperature and in human serum at 37 °C. The radiochemical purity of the complex remained > 98% at room temperature. Also, in the freshly prepared human serum, the same level of purity was observed at 37 °C even after 2 h of preparation.

### Biodistribution of ^68^GaCl_3_ and the radiolabeled complex in Syrian rats

The tissue uptakes of ^68^GaCl_3_ and the radiolabeled complex were calculated as the percentage of the area under the curve of the related photopeak per gram of tissue (% ID/g) (Table 2 & Table 3). ^68^Ga was majorly excreted from the gastrointestinal tract, with high blood content due to transferrin binding at early time intervals. Also, a significant activity content (P≈0.04) was observed in the colon, bone and stomach (maximum values of 1.36 %ID/g, 1.20 %ID/g and 1.54 %ID/g, respectively); however, the kidney was not a significant accumulation site.

**Table 2:** Percentage of injected dose per gram (%ID/g) at 15, 30, 60 and 120 min after intravenously injection of ^68^GaCl_3_ (5.55 MBq) into Syrian rats

Organ	15 min	30 min	60 min	120 min
Blood	3.18±0.17	2.98±0.12	2.53±0.11	2.44±0.14

Kidneys	0.70±0.09	0.41±0.10	1.42±0.12	0.91±0.08

Spleen	0.51±0.09	0.70±0.06	1.03±0.10	1.43±0.12

Stomach	0.41±0.11	0.56±0.07	1.21±0.09	1.54±0.14

Intestine	0.71±0.06	0.82±0.08	0.89±0.05	0.61±0.07

Liver	0.91±0.02	1.50±0.12	0.78±0.08	0.60±0.05

Bone	0.56±0.06	0.91±0.10	1.20±0.11	1.05±0.09

**Table 3:** Percentage of injected dose per gram (%ID/g) at 15, 30, 60 and 120 min after intravenously injection of ^68^Ga-DOTATOC (5.55 MBq) into Syrian rats

Organ	Min 15	Min 30	Min 60	Min 120
Blood	4.51±0.21	1.98±0.15	1.53±0.11	0.18±0.04

Heart	1.68±0.08	1.01±0.07	0.73±0.07	0.03±0.00

Kidneys	11.90±0.21	8.99±0.13	8.00±0.18	2.51±0.10

Spleen	0.38±0.04	0.93±0.07	0.16±0.02	0.12±0.01

Stomach	0.12±0.04	0.18±0.02	0.18±0.03	0.17±0.02

Intestine	0.48±0.06	0.56±0.05	0.39±0.04	0.38±0.04

Lung	0.35±0.06	0.19±0.02	0.13±0.01	0.02±0.00

Liver	0.79±0.09	0.58±0.08	0.46±0.03	0.20±0.00

Skin	0.12±0.03	0.12±0.02	0.08±0.00	0.03±0.00

Bone	0.95±0.08	0.58±0.03	0.42±0.04	0.05±0.00

Muscle	0.40±0.05	0.22±0.02	0.18±0.02	0.02±0.00

Thyroid	0.42±0.02	0.18±0.03	0.16±0.04	0.00±0.00

Adrenal	0.91±0.05	0.75±0.06	0.60±0.04	0.44±0.01

Salivary gland	1.22±0.10	0.77±0.04	0.54±0.03	0.00±0.00

Pancreas	12.83±0.28	9.55±0.18	6.62±0.22	5.31±0.15

The biodistribution of ^68^Ga-DOTATOC demon-strated a significant uptake in the pancreas and adrenal glands (12.83 %ID/g and 0.91 %ID/g, respectively) as the somatostatin receptor-positive organs. The maximum uptake in both organs was reported at 15 min after the injection, showing a slight decrease with time. Kidneys could be considered as a major route of excretion (maximum value of 11.90 %ID/g at 15 min after the injection). No significant accumulation was observed in other organs.

### Imaging studies

**Figure 5:**
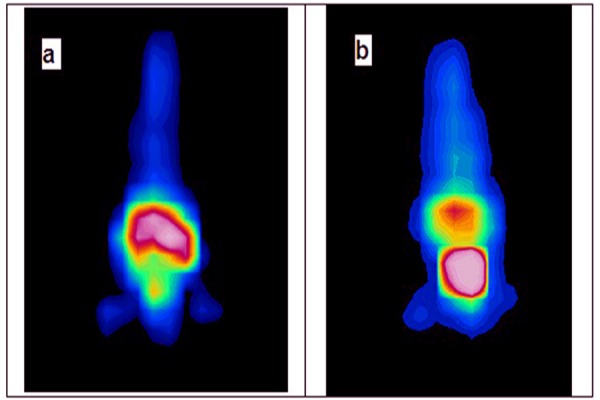
Planar images of Syrian rat at 30 min (a) and 120 min (b) after intravenously injection of ^68^Ga-DOTATOC (5.55 MBq)

Planar images were obtained from normal Syrian rats until 120 h after the radiolabeled complex administration (Figure 5). Imaging of the Syrian rats showed major accumulation of the radiotracer in kidneys and other internal organs such as the pancreas.

### Dosimetric studies

Preliminary dosimetric evaluation in human organs was performed by RADAR method, based on the biodistribution data in rat organs. The absorbed dose was calculated according to DF values in OLINDA software. The absorbed doses in each human organ after ^68^Ga-DOTATOC injection are presented in Table 4. The effective absorbed dose calculated by Equation 6 was 0.026 mSv/MBq.

**Table 4:** Equivalent and effective absorbed dose delivered into each human organ after injection of ^68^Ga-DOTATOC

Target Organs	Equivalent absorbed dose in humans (mGy/MBq)
Adrenals	0.010

Small Intestine	0.008

Stomach	0.003

Heart	0.004

Kidneys	0.074

Liver	0.010

Lungs	0.001

Pancreas	0.104

Red Marrow	0.003

Bone Surf	0.008

Spleen	0.009

Total Body	0.008

## Discussion

^68^Ga-DOTATOC has been applied by several European centers for the treatment of a variety of human tumors (27, 28). Given the use of various ^68^Ge/^68^Ga generators, different techniques for DOTA-conjugated peptides radiolabeling have been developed and employed (29-31). Recently, a SnO_2_-based ^68^Ge/^68^Ga generator was successfully developed in Iran, showing suitable performance for obtaining ^68^Ga-based PET radiopharmaceuticals.

In this study, optimized conditions for ^68^Ga-DOTATOC preparation were obtained, using the newly developed generator. The complex was developed with radiochemical purity of more than 98% and specific activity of 39.6 MBq/nmol (in less than 10 min), which was higher in comparison with previous studies (26 and 11.07 MBq/nmol, respectively) (32, 33).

Several experiments have been performed on the biodistribution assessment of this radiolabeled complex; however, to the best of our knowledge, no data have been reported for time intervals less than 1 hour (2, 34). Besides, the biodistribution of ^68^Ga-DOTATOC has been presented for only some limited organs. In this study, we tried to focus on short time intervals after injection (15 min to 120 min). The biodistribution of this complex was evaluated in numerous organs after intravenous injection in male Syrian rats.

The results demonstrated rapid clearance from blood. Approximately no activity was found in the blood samples after 2 h (0.18 %ID/g). As expected, significant uptake was observed in somatostatin receptor-positive tissues such as pancreatic and adrenal tissues, which decreased slightly with time (5.31 %ID/g and 0.44 %ID/g at 120 min after the injection, respectively).

Considerable aggregation was indicated in the kidneys as the major excretion route (11.90 %ID/g at 15 min after the injection); this finding was in accordance with previous literature. However, no considerable accumulation was detected in other organs. Specific uptake and retention in the target organ, rapid clearance from blood and negligible uptake in non-target organs turned ^68^Ga-DOTATOC into an ideal imaging agent.

Although substantial clinical data (>1000 patients) have asserted the safety and efficacy of ^68^Ga-DOTATOC, definitive dosimetric data are yet unavailable (35, 36). Studies have been performed to calculate the absorbed dose of this novel agent, although significant discrepancy has been observed in the reported data.

In this regard, in a study by Ugur et al., the absorbed dose of kidneys was calculated to be 1090 mGy/MBq, based on biodistribution studies on AR42J rat pancreatic tumors (2), whereas an absorbed dose of 0.082 mGy/MBq was reported in kidneys of patients with disseminated neuroendocrine tumors (37). However, the absorbed dose of the pancreas as a somatostatin receptor-positive organ was not evaluated in previous research.

A prerequisite for the clinical application of new diagnostic radiopharmaceuticals is the measurement of organ radiation exposure dose, based on biodistribution data in animals (38). These results can be used to estimate the maximum permissible administered activity, which maintains the organ doses within an acceptable range. Overall, minimizing radiation exposure to patients, while providing scintigraphic images with suitable quality, is the major purpose of imaging procedures.

Although the extrapolation of animal data to humans may lead to over- or under-estimation, previous studies have indicated the usefulness of animal biodistribution models for absorbed dose estimations in humans (39). This is in fact a common first step, consistent with the recommendations of ICRP publication 62 (40).

In this study, we tried to estimate the equivalent and effective absorbed doses in numerous human organs with higher accuracy, considering the biodistribution data in normal rats within short intervals after the injection.

In the present study, maximum absorbed doses were reported in the pancreas, kidneys and adrenal glands (0.105, 0.074 and 0.010 mGy/MBq, respectively). Our findings were more consistent with the results reported by Sandstrom et al., compared to the study by Ugur and colleagues.

As mentioned earlier, the dose delivered to the pancreas has not been evaluated in previous literature. However, in our study, the absorbed dose of kidneys (0.074 mGy/MBq) was comparable with the value reported in the study by Sandstrom et al. (0.082 mGy/MBq) (36). On the other hand, the absorbed dose of adrenal glands was somewhat lower than the value reported in the mentioned study, while being in great accordance with the absorbed dose of liver after ^68^Ga-DOTATATE injection (0.015 mGy/MBq) in a study by Walker et al. (41).

In the present study, the effective absorbed dose was calculated at 0.026 mSv/MBq, which was higher than the value reported by Sandstrom and colleagues (0.021 mSv/MBq). The lower effective absorbed dose in the mentioned study corresponded to lower organ consideration for dosimetric calculations, compared to the present study. Finally, the dose received by critical organs was well within the acceptable range for diagnostic nuclear medicine procedures (31, 42).

## Conclusion

In this study, ^68^Ga was obtained from a recently developed generator in Iran. The results of quality control analysis including the assessment of radionuclidic, chemical and radiochemical purities indicated that the eluted ^68^Ga had desirable characteristics and could be used for radiolabeling purposes. ^68^Ga-DOTATOC was prepared with radiochemical purity of more than 98% and specific activity of 39.6 MBq/nmol. The complex demonstrated great stability at room temperature and in human serum at 37 °C at least for 2 hours. Significant uptake was observed in somatostatin receptor-positive tissues (e.g., pancreatic and adrenal tissues). Maximum absorbed doses were found in the pancreas, kidneys and adrenal glands (0.105, 0.074 and 0.010 mGy/MBq, respectively). Also, the effective absorbed dose was 0.026 mSv/MBq for ^68^Ga-DOTATOC. The results indicated that the newly developed ^68^Ge/^68^Ga generator has the potential to prepare peptide-based radiopharmaceuticals. Also, the biodistribution study of ^68^Ga-DOTATOC showed desirable outcomes. The absorbed dose of this complex by critical organs was well within the acceptable range for diagnostic nuclear medicine procedures, which was comparable with previous literature. Therefore, ^68^Ga-DOTATOC can be considered as an effective agent for clinical PET imaging in the country.
